# The value of health care – a matter of discussion in Germany

**DOI:** 10.1186/1472-6963-7-1

**Published:** 2007-01-02

**Authors:** Franz Porzsolt, Moritz Ackermann, Volker Amelung

**Affiliations:** 1 Clinical Economics, University of Ulm, Germany; 2 Department of Epidemiology, Social Medicine and Health Systems Research, Hannover Medical University, Germany; 3 Federal Association of Managed Care, Berlin, Germany

## Abstract

**Background:**

Interest in assessing the value of health-care services in Germany has considerably increased since the foundation of the Institut für Qualität und Wirtschaftlichkeit im Gesundheitswesen, IQWiG (Institute for Quality and Efficiency in Health Care). The practical application of value assessment illustrates how problematic the process can be. In all decisions made for the provision of health care, data concerning the measurable dimensions (quantity and quality of efficacy and effectiveness, validity of the results and costs) flow into a complex and not yet standardized decision-making process concerning public financing. Some of these decisions are based on data of uncertain validity, unknown reproducibility and unclear appropriateness.

**Discussion:**

In this paper we describe the theoretical aspects of value from psychological and economic viewpoints and discuss national and international approaches. Methodic details and difficulties in assessing the value of health-care services are analysed. A definition of the intangible value of health-care services will be proposed which contains only three factors: the absolute risk reduction (usually a measure of efficacy), the validity of the scientific papers examined and the type of the expected effectiveness (prevention of death and disability, restitution of well-being). The intangible value describes the additional benefit when comparing two possible actions, like treatment or observation only.

**Conclusion:**

The description of intangible value from the viewpoint of different stakeholders is a useful measure for subsequent steps (not discussed here) – the evaluation of costs and of patient benefit. A standardised, transparent, fair and democratic evaluation is essential for the definition of a basic benefit package.

## Background

The foundation of the Institut für Qualität und Wirtschaftlichkeit im Gesundheitswesen, IQWiG (Institute for Quality and Efficiency in Health Care) exemplified the need for a scientific and independent evaluation of health-care services in Germany. Since a basic benefit package should be publicly financed, the assessment of its value cannot rely merely on supply and demand. The problem arises due to the different points of view concerning what is adequate, appropriate and economic. The definition of this range of services is a political decision which should be supported to the greatest possible extent by scientific data.

For this purpose, institutions have been established in different European states and in Australia and Canada to carry out a scientific evaluation of the available data [assessment] and then to perform a political interpretation and make decisions [appraisal]. In most countries, these two steps – assessment and appraisal – are performed by separate and independent institutions. The pioneering function of Anglo-Saxon countries is plausible because the necessity of making transparent decisions in their health-care systems, which are financed by the national treasury, is considerably greater than in systems which are based on private and social insurance systems.

In most industrialized countries, health care is one of the most important market segments and, therefore, has considerable importance for the overall economy. Appropriate growth of this market and a high potential for innovation can be considered positive indicators for the welfare of a nation. The market and its innovations can hardly be controlled by uniform viewpoints if the value of its products cannot be measured. Without knowing the values of a culture, the appropriateness of prices can hardly be judged. Our considerations on this subject have been summarized in two books, 'Klinische Ökonomik' (Clinical Economics) [1] and 'Optimizing Health – Improving the Value of Healthcare Delivery' [2].

The aim of this paper is to propose and discuss procedures to assess the value of publicly-financed health-care services. The procedures must be reliable and valid and should be applicable in all health-care systems. To achieve the defined goal, theoretical aspects of value are discussed from economic and psychological points of view, and the problem of the general measurability of value is addressed.

## Discussion

### Concepts of value

As various disciplines investigate different aspects of value assessment, a short theoretical excursion may be helpful in the discussion of a new concept.

### Economic value

The classics of economic theory apply an objective, cardinal term for value which is oriented to the recognized value of a commodity for the satisfaction of specific needs, like the caloric value of coal or the highest permitted speed of a car. The neoclassical theory of value, in contrast, proceeds from an ordinal, subjective concept of value in which an absolute measurability of value is rejected in favour of individual hierarchies of preference [3].

### Psychological value

In the field of psychology, value is defined as subjective well-being and divided into different dimensions. Pleasant emotions, unpleasant emotions and cognitive life satisfaction are differentiated [4].

### Operationalisation

#### Definition of value

In economic theory, value is understood as the measurement of the ability of a good or an object to satisfy the needs of an economic stakeholder (like a consumer). Need is defined as the attempt to relieve discomfort through a certain action. This concept is also applied in the classic definition of quality in DIN ISO 9000:2000.

#### Types of value

The households of microeconomic consumption theory are maximisers of value in their consumption demands. They measure the quality of consumed goods as the degree of received value.

The contributions of the school of marginal costs (the value of an additional unit of a good) and the introduction of the term 'subjective utility' as an individual assessment of a good as an exchange or utilitarian object by the evaluator led to the identity of utilitarian or exchange value.

Households, as well as users of health-care services, behave rationally in that the overall value of the consumed goods is as high as possible under the budgetary limitations of the available income. In public health-care systems, the users of health-care services also behave rationally in that the overall value of the consumed goods is as high as possible, but not – and this makes the difference – limited by their own income. The users try to get "unlimited health care". As a consequence, not the users of health-care services, but others, have to make the decisions about the allocation of health-care resources. These proxy decisions are rather difficult to make due to the difficulty of measuring the value of health care.

#### Measurability of value

The usual techniques applied by economists [5-8], like standard gamble and time trade off, are excellent for theoretical considerations, but cannot be applied to daily clinical practice. Ten minutes of the doctor's full attention and the perception of empathy are much more valuable to patients than twenty business minutes of the doctor's time budget. In other words, a formal expression of the value of health care may be plausible for policy considerations of health-care managers, but not for individual patients who have a serious problem.

The standardized assessment of quality of life or of well-being is obviously problematic because each individual does not measure his/her received value on these scales, but in other terms. This has recently been shown in patients with different types of cancer. These patients exhibit different individual preferences for health-related quality of life [9]. Breast-cancer patients under treatment wish to avoid nausea/vomiting, pain and a decrease in emotional and role functioning. Patients with colorectal cancer listed nausea/vomiting and diarrhoea before pain and role functioning, and for patients with lung cancer, it was more important to avoid dyspnea than other symptoms.

#### International assessment of health-care services

A synopsis of the procedures used in different countries carried out for the Deutsches Institut für Medizinische Dokumentation und Information, DIMDI (German Institute for Medical Documentation and Information) provides a comprehensive description of the complexity of decision-making processes, the institutions involved and the methods used to make these decisions [10]. Without going into the details of the analysis, it becomes clear that the complex process of evaluation must eventually lead to a one-dimensional decision – whether a health-care service is to be fully, partly or not publicly financed. Even this carefully performed analysis cannot represent the applied procedures with sufficient clarity to make them comprehensible.

Far more problematic than measurement is the assessment of these results. It is not described anywhere in the included value assessments which criteria of value, like ameliorating chronic complaints in degenerative diseases, are compared with the value of a therapy which could prevent the late results of a disease if compliance is high. How scientific papers describing important clinical effects (like prevented deaths) not supported by valid data are evaluated remains unclear.

American authors [11] requested 225 leading internists to estimate the importance to patients of 30 medical interventions. The experiment took the inevitable subjective viewpoints of the assessors into consideration. Although different aspects of this procedure are open to criticism, the procedure appears to indicate how a consensus on useful results can be reached.

Unfortunately, similar uncertainties observed in effects are also reflected in costs. The critical evaluation of 89 cost-utility ratios from 40 oncology studies demonstrates that the validity of many of these examinations are questionable when carefully analysed [12].

### National assessment of health-care services

It is probably no coincidence that the term 'value' is often addressed, but not explicitly stated anywhere in the German Drug Law. This law defines pharmaceutical drugs as substances or preparations from substances with which physical or mental suffering can be cured, lessened, prevented or recognized. A generally accepted definition of the value of health services would also prove helpful here.

The legal foundation for the assessment of the value of a drug can be found in the Sozialgesetzbuch [Code of Social Law]. Paragraph 1 (V, §35b) states that the Institute for Quality and Efficiency in Health Care determines uniform methods to establish the evaluation of value and publicises them in the internet. Paragraph 2 describes the testing and, if necessary, alteration of these methods.

A rational examination of scientific criteria can only be carried out on the basis of a scientific discussion. Therefore, scientific suggestions stemming from different national institutions for assessing the value of health-care services will be summarised in the appendix.

Both psychology and economics offer several theoretical approaches to define the value of health care, but health-care professionals are not satisfied with either of them, and it seems they are not applicable. Economics reduces the economic stakeholder, in simple terms, to a purely economic being who is driven solely by self-interest and behaves purely according to rational criteria. One of the core assumptions is the principle of marginal costs and marginal benefit. This concept does not add value if the choice is only being alive or dead. Another problem emerges when life-threatening situations are implicitly considered in discussions about the value of health-care services, neglecting the large variety of services ranging from rescue to cosmetic interventions.

The "homo economicus" desires to equalise the marginal value of his/her various consumer possibilities and, thereby, to maximize his/her overall value. The desire for value among all members of society creates a demand for goods, which determines the supply structure via the market-price mechanism. The market-price mechanism itself depends on limitations, such as scarcity of goods and the available budget. As there is no real limitation on many "goods" (except rationing) in the health-care market as long as the public pays for them, an essential control mechanism is lacking in publicly-financed health care. The laws of economics will be more effective in a privately-financed health-care sector. The critical solution needed for all health-care systems is the assessment of value in the public sector.

The meaning of value in psychology, with its goals of happiness and well-being, more closely resembles the opinion of sick persons than the economic concept. The meaning of death for terrorists is completely different from that of chronically-ill persons. In medicine, the concept of benefit or value is not standardised, which can be explained by the lack of a theory of medicine [13]. Because medicine is an empirical science without a theoretical basis (bio-molecular foundations cannot replace a theory of medicine), uniform definitions of health, sickness and the value of health services are lacking. This lack of a fundamental theory explains the reluctance of physicians to discuss theoretical aspects of medicine.

Many physicians also feel uncomfortable with the methodic requirements of clinical epidemiology unless psychological aspects of daily medical practice are considered. Clinical science is impossible without stringent epidemiologic methods.

• Almost all of the data used in cost-benefit analyses derive from clinical studies, which assessed the efficacy, but not effectiveness (effects under everyday conditions) of treatment. This problem refers to the interface of clinical epidemiology and economics.

• The methods used in economic analyses (like "time trade-off" – how many years of life would you be willing to forfeit if you could exchange your present health condition for optimal health) do not, in contrast to the opinions of some economists, have anything to do with clinical reality in our culture. Most patients value life and struggle for each day they can remain alive. This is a problem at the interface of psychology and economics.

• The last statement is supported by data which demonstrate that patients generally place a high value on life, regardless of its quality [14,15]. In quality-of-life research the phenomenon of "response shifts" is known. Self-assessed quality of life describes the relationship between the individual's expectations and observations. Most patients report the same quality of life even during progression of their disease associated with an increase in problems and impairments. The most likely explanation for this observation is the adaptation of the patient's expectations to reality. Lowered expectations correspond with the observed situation, resulting in a stable quality of life.

• The generic instruments for quality-of-life assessments preferred by economists (comparing outcomes in different diseases) are too insensitive to detect small, but significant, treatment effects. Therefore, psychologists develop highly specific questionnaires to detect aspects that are important to patients. Physicians who are socialised in a bio-molecular world disregard the "soft" research conducted by psychologists and prefer to base their decisions on "hard facts", such as laboratory results and imaging methods, that are often irrelevant to patients and of unknown prognostic significance.

It may seem strange that most of the studies and presentations cited in the appendix of the national assessment of health-care services were directly or indirectly initiated by the pharmaceutical industry. One reason is that the proof of effectiveness of drugs is one of the business foundations of the pharmaceutical industry and that experience with such instruments and methodic expertise are available there. Unfortunately, the purchasers of health-care services are often unfamiliar with the theories behind their decisions. Values from the viewpoint of different stakeholders are usually not taken into consideration.

To bridge this gap we introduce the term "intangible value" of health-care services. This concept combines aspects of clinical epidemiology and psychology to form a basis for necessary economic assessments. Value is considered as an *additional benefit *obtained by possible alternative actions. Even if there appears to be only one possible mode of action or therapy, there are always the two possibilities of either applying the existing option or not. Reasons for not applying it could be undesired side effects, like high monetary costs or impairment of quality of life.

To reach a consensus on the definition of the value of health-care services, we assume that the value relevant for making a decision coincides with the subjectively perceived value. The actually achieved value influences the perceived value, but is not relevant for making a decision. Most decisions reached in the health-care system cannot be explained by considering only the actually confirmed value. If 1000 women undergo mammography within a time interval of 10 years, death as a result of breast cancer can be prevented in one woman. About 970 of these 1000 women receive the anticipated information following the examination that no adverse finding could be detected. One can now pose the question whether this examination, which is important to many women, is largely demanded due to the *perceived *or the *actually guaranteed *safety [16].

The term 'intangible value' in analogy to the term 'intangible costs' is used to illustrate that the concept of subjectively perceived value used here is considered independent of monetary costs. The intangible value of a health-care service coincides with the added value which is perceived to be provided by the new service in comparison to already existing services. This intangible value is defined by three dimensions:

• the effects expressed as absolute risk reduction (ARR),

• the validity (V) of epidemiologic and of economic data and

• the type of expected effectiveness (TEE) (saved life or alleviated complaints under day-to-day conditions).

The parameters measured as endpoints of clinical studies, like prolongation of survival, improvement in quality of life, lowering of high blood pressure, changes that can be identified in imaging procedures, undesired side effects of drugs, and increased compliance, are considered "effects". Effects are expressed as absolute risk reduction (ARR). This describes the number of patients that have to be treated to observe a specific problem (e.g., death, recurrence of malignant disease, or headache) in the experimental-treatment group in one case less than in the control group.

The collection and description of the data (assessment) in these three categories, ARR, V and TEE, require specialist knowledge. Experts are superior to laypersons in the assessment. Specialists, however, are less adept in the interpretation (appraisal) of the measured data because the interpretation is made according to subjective (!) criteria.

### Absolute risk reduction (ARR)

Evidence-based medicine groups in Hamilton, Ontario (Canada) and Oxford (England) were very effective in disseminating the rules of clinical epidemiology and other helpful principles for medical decision making. Therefore, it can be anticipated that medical students are familiar with the concepts of risk reduction.

### Validity (V)

To avoid subjective, arbitrary evaluations, we propose letting all groups which participate in the value assessment evaluate the validity. This requires that all members of these groups have a basic knowledge of epidemiology, which can be attained quickly [17,18] and with an acceptable amount of effort. Aside from the studies and meta-analyses cited above, which support the view that the role of randomisation is overestimated, the apparently unproblematic differentiation between randomised and non-randomised studies could contribute to the overestimation of this criterion of validity. A further criterion of validity, blinding/masking, can be just as important as randomisation. Preventing unmasking, which is as important as masking, is considerably more difficult to maintain and to identify in a published study than the difference between a randomised and a non-randomised study. This example shows that the validity of scientific statements can only be confirmed by paying attention to details.

Even in qualitatively well performed meta-analyses of treatment studies the validity is not formally taken into consideration when calculating the effect. In other words, studies with low validity – if they are not excluded by the authors of meta-analyses due to considerable deficiencies – influence the result of a meta-analysis to the same degree as valid studies. In nearly all evaluations, it is, however, the importance relegated to the published data is decisive in the decision-making process. Studies in which very big effects are described contain a priori a higher validity risk than studies in which small effects are described.

### Type of expected effectiveness (TEE)

An unsolved problem is how realistically the results of controlled studies which have been conducted under ideal conditions (e.g. a randomised controlled trial) can actually be applied to everyday conditions. It is almost impossible to maintain the inclusion and exclusion criteria and other conditions prevailing in a controlled study under everyday conditions. Maintenance of these conditions in a controlled study is, however, essential to be able to confirm the described effects with scientifically recognized methods. It is also hardly possible to realistically demonstrate "minor" improvements in the quality of care with the traditional methods of clinical studies. By minor improvements we mean, for example, modifications which improve compliance. It is easy to imagine that an improvement in patient compliance is unimportant in a clinical study because close controls take care of the problem of compliance. Under everyday conditions, however, improved compliance can exert a considerable influence on outputs and outcomes. Therefore, the classification as a "minor" advantage may simply be wrong.

Evaluations, for example, of an improvement in hearing, childlessness, false teeth, or innovative therapies for incurable diseases are problematic and are made primarily by decision makers. The perceived suffering is correlated with the recognised value of the therapeutic measure. The greater the suffering, the higher the value attributed to the appropriate treatment. Non-affected (healthy) laypersons are probably not inferior to experts in making these decisions because experts cannot avoid their specialty biases [19,20].

In summary, the data of the three measurable dimensions (effect expressed as ARR, validity, and type of expected effectiveness) are subject to complex and hitherto incomprehensible processes which result in a decision concerning public financing. The rules for these decisions have to be defined to guarantee consistency and reproducibility. To our understanding, the term "intangible value" includes multidimensionality (i.e., several product characteristics are taken into consideration, like therapeutic and non-therapeutic, objective and subjective criteria, desired and undesired effects), complexity (i.e., evaluated from different perspectives, like patient [intended or unwanted effects], physician [effectiveness], health insurer [efficiency], transparency (i.e. differentiation between objective assessment and subjective interpretation) and time dependency (i.e., the expected effect can be delayed and is temporally limited).

Finally we propose an emerging model for assessment of the "intangible value" to be applied by decision makers representing the important stakeholders in a health-care system, e.g., 37.5% recipients of health-care services (insured individuals), 37.5% providers of health-care services, (i.e., 25.0% specialist societies and 12.5% industry), 15.0% public payers of health-care services (insurances), and 10.0% independent experts (scientists).

These members may be appointed for a limited period (like 4 years) and replaced every half year. The procedure for choosing members within the individual groups should reflect the actual spectrum of the insured individuals, service providers, and payers of health-care services.

After short training in epidemiologic foundations of the evaluation of scientific data, this panel will present the health services which are to be evaluated. The panel members are able to understand scientific data on effect, effectiveness and validity of data and are aware of the limitations of objective evaluation. The data for the effects and validity will be presented to the panel members by "independen" scientists (as far as they can be independent, e.g. from NICE in the UK or IQWiG in Germany). Based on this presentation the panel members interpret three dimensions of the presented data – the absolute risk reduction (ARR), the validity (V), and the type of expected effectiveness (TEE) – by using a score point system (Table 1) which is subject to validation.

**Table 1:** Calculation of the "intangible value"

Dimensions influencing the "intangible value"	Comparison between effects of two treatment options	Results based on scientific result or consensus
Absolute Risk Reduction [ARR]	ARR>40%:10 pts; 39-25%: 9.9-9.0 pts; 24-15%: 8.9-8.0 pts, 14-10%: 7.9-7.0 pts; 9-5%: 6.9-6.0 pts; ARR<5%: 5 pts	Absolute risk reduction (ARR) = 14% 7.6 points
Validity [V]	Validity (multiplier for not impaired validity = 1.00, for seriously impaired validity = 0.10)	Subjectively determined validity factor: 0.90
Type of expected effectiveness [TEE]	Type of expected effectiveness (TEE) (multiplier for prevention of: death = 10.0; life-threatening event = 9.9-9.0; considerable disability = 8.9-7.0; dis-ability = 6.9-4.0; disturbance in well-being = 3.9-1.0	Subjectively determined type of exp. effectiveness (TEE) = 9.2
	Intangible value (product of ARR score) × (validity factor) × (TEE)	Intangible value = 7.6 points × 0.90 × 9.2 = 62.9 points

The difference in effects (absolute risk reduction) is expressed in score points. These points are multiplied by a validity factor and factor describing the type of expected effectiveness. The given example assumes an ARR = 14%, high validity (0.90) und important type of expected effectiveness (9.2). The calculated intangible value is 62.9 points.

There will not be much variation in the interpretation of the efficacy, which is usually expressed as absolute risk reduction (ARR) [21,22]. A maximum of ten points can be reached for ARR > 40%. The range of 9.9 – 9.0 points is proposed for ARR = 39%–25%; 8.9 – 8.0 for ARR = 24 – 15%; 7.9 – 7.0 points for ARR = 14 – 10%; 6.9 – 6.0 points for ARR = 9 – 5% and 5 points for ARR < 5%.

In contrast, the interpretation of the validity of scientific reports varies considerably. It has recently been shown that application of the criteria of the CONSORT statement improves the reporting of clinical trials [23,24]. Unfortunately, these recommendations [25-28] are not always accepted [29]. A more general problem is the inclusion of clinical trials in systematic reviews. Although there are recommendations for the inclusion or exclusion of trials [30] in systematic reviews, the impact of trials included in a systematic review is generally independent of its validity. We consider this to be a serious problem, as the chance to produce the expected results in experimental studies increases with decreasing validity of the studies. Therefore, we propose incorporating the validity (V) of the analysed studies in our model. This is achieved by multiplication by a simple validity factor which is 1.0 if the validity of a study is not impaired at all. This factor may be as low as 0.1 in studies with seriously impaired validity. Further details are shown in Table 2.

**Table 2:** Suggested factors (range 1.0 – 0.1) for evaluation of the validity [V] of scientific papers and the assessment of type of expected effectiveness [TEE] (range 10.0 – 1.0).

Factor	Validity	Type of expected effectiveness (TEE) × 10 (prevention of ...)
1.00	Not impaired	Death
0.99 – 0.90	Almost not impaired with few deficiencies	life-threatening event
0.89 – 0.80		considerable impairment
0.79 – 0.70	impaired with deficiencies	
0.69 – 0.60		
0.59 – 0.50	clearly impaired with obvious deficiencies	
0.49 – 0.40		Impairment
0.39 – 0.30	rather impaired with considerable deficiencies	
0.29 – 0.20		
0.19 – 0.10	seriously impaired	disturbed well-being

TEE describes the importance of a health-care service preventing either death, disability, or disturbance in well-being under day-to-day conditions. The International Classification of Functioning, Disability and Health (ICF) describes the foundations of this evaluation.

The third variable which has to be included in the score of intangible value is the type of expected effectiveness (TEE), which actually inccorporates two aspects. First, it differentiates between treatments that are life-saving (TEE = 10.0) or just avoid disturbances in well-being (TEE = 3.9 to 1.0). A more detailed proposal for assessments is shown in Table 2. The second aspect which is included in the TEE value is the difference between efficacy (assessment under ideal conditions) and effectiveness (assessment under everyday conditions). Data that describe the relationship between efficacy and effectiveness are lacking. As treatment effects are usually assessed under ideal conditions, i.e. the efficacy, but not the effectiveness, is assessed, we can describe their expected (type of expected effectiveness), but not their real effectiveness. The TEE value therefore includes the estimated difference between efficacy and effectiveness.

The panel should only evaluate health services for which a discussion of public financing by authorized institutions is expected. A two-step procedure can be performed to attain the expected added value of these health services.

First step: The attainable scientific data on which the comparative evaluation of at least two procedures is based (database) are elucidated. A date for the presentation of the question and the preliminary data is set with the panel. The database is made available to the members of the panel. Scientists present the essential data which describe the efficacy or effectiveness of the examined service, as well as the validity of the studies examined. The panel should have sufficient time to comprehend the presented results based on the available documents and to formulate complementary questions.

Second step: Within three weeks a second step is initiated. At this time the members of the panel ascertain in a written procedure (the voting person is not identified, but the group to which s/he belongs is) the intangible value as described above.

Even this simplified evaluation can be based on very complex considerations. In 2001 the WHO presented the "International Classification of Functioning, Disability and Health (ICF), which is analogous to the International Classification of Diseases (ICD). This classification does not offer a quantitative evaluation because this would be subject to considerable variation due to the subjective points of view. The ICF lists all aspects which should be considered in the complex evaluation of the performance category.

The resulting intangible value has to undergo a validation process. The proposed model which takes the intangible value into consideration might be a first step towards a synthesis of aspired goals defined by health-care practitioners and methods contributed by economists.

### Summary

The ongoing discussion in Germany about the value of health care and its public financing triggered this summary of existing models for assessment of the value of health care. We address some limitations of these models and propose a method that is based on three variables: the efficacy of the service, the validity of the scientific reports and the type of expected effectiveness. The last variable includes two estimates – the value of alleviation of a symptom or the prevention of death and the probability that the effect which was measured under the ideal conditions of a controlled clinical trial will also occur under everyday conditions. The (not yet validated) product of these three variables provides a figure that can be used for comparisons and may be useful for the definition of a basic benefit package.

## Abbreviations

ARR Absolute risk reduction

BAH Bundesverband der Arzneimittelhersteller (Federal Association of Drug Manufacturers)

BKK Betriebskrankenkassen (Company-affiliated social health insurances)

BPI Bundesverband der Pharmazeutischen Industrie (German Pharmaceutical Industry Association)

DIMDI Deutsches Institut für Medizinische Dokumentation und Information (German Institute for Medical Documentation and Information)

DPhG Deutsche Pharmazeutische Gesellschaft (German Pharmaceutical Association)

IQWiG: Institut für Qualität und Wirtschaftlichkeit im Gesundheitswesen (Institute for Quality and Efficiency in Health Care).

TEE Type of expected effectiveness

V Validity

VFA Verband der Forschenden Arzneimittelhersteller, (German Association of Research-Based Pharmaceutical Companies)

## Authors' contributions

FP drafted the manuscript. MA and VA contributed the aspects of economics and business economics. All authors read and approved the final manuscript.

## Appendix

Scientific suggestions for assessment of the value of health-care services

A position report of the Betriebskrankenkassen, BKK (Company-affiliated social health insurances) [31] established that the effectiveness according to the Arzneimittelgesetz (German Drug Law) corresponds with the socio-legal term "expediency", i.e., with the therapeutic value of a drug. The service obligation is to be decided based on cost-benefit considerations. The criteria for the list of recommended drugs states that the therapeutic value can be measured by the extent to which the desired therapeutic effect is attained. This observation is important from a scientific point of view, but the application of different evaluation criteria for chemically defined and other drugs (e.g., from the fields of phytotherapy, homeopathy, anthroposophy) is problematic. According to this definition, the therapeutic value of chemically-defined drugs must be investigated according to evidence-based criteria while "other" drugs only have to comply with internal evaluation systems founded on scientific theory.

A position paper [32] of the Verband der Forschenden Arzneimittelhersteller, VFA (German Association of Research-Based Pharmaceutical Companies) presented a detailed suggestion for different criteria which should be observed in the assessment of the value of drugs. It stated that the assessment of value should exceed the boundaries of individual sectors, i.e., should include the prevention or reduction of hospitalisations, early retirement, nursing care or visits to physicians. An appropriate point in time should be chosen for the assessment of value, i.e., the indication and other circumstances determine how great the time interval between registration and possible evaluation of value should be because statements concerning the value of a new therapy immediately following its registration are, by definition, not possible. Registration usually results from proof of efficacy, rarely of effectiveness, and almost never of value. The value of a drug or a health-care service certainly cannot be sufficiently described by its efficacy or effectiveness. Therefore, we point out the necessity of a scientific differentiation between efficacy, effectiveness and value.

It is also correct that the methodic approach to assessing value should not be too narrow. The use of only randomised studies – as suggested by German authorities – to describe the value can hardly be justified scientifically because these studies have both advantages and disadvantages, just like any other scientific method. Since the appropriate definition of evaluation criteria represents one of the greatest problems in assessing value, one must pay particular attention to maintaining scientific criteria. Arbitrary rules including minimum times or minimum dimensions cannot be upheld from a scientific standpoint. Most complex decisions require a structured dialogue, which we confirm to be an essential part of value assessment.

In its final report on short-acting insulin analogues, the Institute for Quality and Efficiency in Health Care demands observation of long-term effects to evaluate benefit and harm [33]. This requirement makes sense if it can be fulfilled under the prevailing conditions [34]. Since the required data have not yet been collected and cannot be handed in within a short time, other ways to assess value must be discussed.

The report also mentions additional benefit relevant to the patients [33], but provides no indication of what is meant by the term additional benefit and with which data this should be proven. Thus, the statements made by the Institute for Quality and Efficiency in Health Care do not make the anticipated contribution to the evaluation of health-care services.

This impression has been confirmed in a critical statement [35] concerning the commission and the activities of the Institute. It has also been accused of a lack of independence and unscientific arguments. This reproach is not unjustified, since the practicability of generally accepted assessment rules has been questioned without mentioning the available literature on the term value.

The Deutsche Pharmazeutische Gesellschaft, DPhG (German Pharmaceutical Association) contributes to the definition and differentiation of different forms of innovation in its position paper [35]. When referring to innovation, "(additional) value" is pointed out along with the "(real) innovativeness", whereby the former usually can only be evaluated some time after an innovation has been introduced. In this case, it would be reasonable to name the criteria and the expected time for the confirmation of value as soon as an innovation is recognised as such.

A comprehensive description of value assessment containing a summary of the discussed aspects was established by the Bundesverband der Arzneimittelhersteller BAH e.V. (Federal Association of Drug Manufacturers) [37]. Therein from an economic viewpoint value is understood as an abstract measurement for the satisfaction of needs which a consumer can get from consuming a good. Value is equated with health-related quality of life and is, therefore, subjective and related to time and place.

This concept is, however, problematic in practical application because:

• value must be measurable (objective, interpersonally comparable and it should be possible to aggregate it as social benefits),

• value must reflect different perspectives and

• the conflict between individual and social value has to be solved.

It is shown that socio-legal aspects (expediency, efficiency, not surpassing the required limits) are necessary, but not sufficient, criteria for value assessment. Criteria of welfare economics and regulatory policies can hardly be applied for value assessment because the physician's decisions have to be tailored to the individual patient. Even if it hardly seems possible to take these three aspects into consideration in a common strategy for value assessment, an approximate solution which is supported by a sufficiently large majority remains a worthwhile goal in the absence of other reasonable alternatives.

The Bundesverband der Pharmazeutischen Industrie, BPI (German Pharmaceutical Industry Association) [38] illustrated the value assessment of drugs under methodic aspects with ten examples. These examples describe the complexity of value assessment by demonstrating the measurement of effectiveness, quality of life, compliance, side effects and costs on different models. The introduction to the topic contains a general and technically well-founded criticism of methods relating to value assessment in medicine. This presentation of the components of value assessment is a prerequisite for the following necessary step of finding a consensus in which these components are summarised into a value judgment for an indication and a treatment method.

The drug manufacturer, Pfizer Germany, [39] recruited scientists from different disciplines and institutions for a pertinent discussion of the value of drugs. The didactically valuable, comprehensible contributions reflect the different understanding of the various methods which are applied to describe value in the different disciplines. The contributions appear to coincide in the opinion that a differentiated judgment of the benefit for the individual patients in everyday medical care is not possible. We are not yet satisfied with this result because, when there is no consensus on the value assessment, there is a risk that arbitrarily chosen criteria will be legitimated either to publicly finance health services which are of little patient benefit or not to publicly finance beneficial services and, thereby, restrict their availability to part of society. For that reason we are strongly in favour of gaining a consensus concerning the value assessment of health-care services. Even if this consensus initially has severe limitations, it makes more sense to begin the process of consensus building now than later.

Perleth [40] and Busse [41] called attention to the growing importance of Health Technology Assessment (HTA) Reports in the evaluation and reimbursement of health-care services. Although the importance of these summary reports is generally accepted, one must keep in mind that there exist qualitative differences among these reports. If the quality of scientific papers which are summarized in HTA reports reveals errors and these are not mentioned in the HTA report [42], a major pillar of our decision-making processes could become instable. The compilation of complex data with scientific methods does not necessarily increase the reliability of their statements. In our own interest we should consider whether quality-ensuring measures should be introduced for these important components.

## Pre-publication history

The pre-publication history for this paper can be accessed here:


